# Clinical outcomes of tisagenlecleucel in relapsed/refractory diffuse large B-cell lymphoma: insights from a single-center study

**DOI:** 10.1007/s12185-025-04006-z

**Published:** 2025-08-22

**Authors:** Sang Eun Yoon, Junhun Cho, Duck Cho, Eun-Sook Kang, Seok Jin Kim, Won Seog Kim

**Affiliations:** 1 Division of Hematology-Oncology, Department of Medicine, Samsung Medical Center, Sungkyunkwan University School of Medicine, 81, Irwon-Ro, Gangnam-Gu, Seoul, 06351 Korea; 2 Department of Pathology, Samsung Medical Center, Sungkyunkwan University School of Medicine, Seoul, Korea; 3 Department of Laboratory Medicine and Genetics, Samsung Medical Center, Sungkyunkwan University School of Medicine, 81 Irwon-Ro, Gangnam-Gu, Seoul, Korea

**Keywords:** Chimeric antigen receptor T cell therapy, Tisagenlecleucel, Cytokine release syndrome, Immune effector cell-associated neurotoxicity, Relapsed/refractory diffuse large B-cell lymphoma

## Abstract

Chimeric antigen receptor (CAR) T-cell therapy has transformed the treatment landscape for relapsed and refractory large B-cell lymphoma (RR-DLBCL). This study evaluated the real-world efficacy and toxicities of 96 patients with RR-DLBCL who received tisagenlecleucel (tisa-cel) at a single institution. Among 81 patients who received bridging chemotherapy, most received a bendamustine and rituximab regimen (*n* = 48, 46.9%), and 31 (38.3%) responded to bridging chemotherapy (17.3% complete remission [CR] and 21.0% partial remission). Tisa-cel showed an overall response rate (ORR) of 71.9% at 1 month, which declined to 55.1% at 3 months. The median progression-free survival (PFS) was 4.5 months, with 1-year PFS at 33.3%. Median overall survival (OS) was 13.9 months, with a 1-year OS rate of 55.2%. Achieving CR at 3 months was correlated with significantly improved PFS and OS. Cytokine release syndrome occurred in 75% of patients (14.6% grade ≥ 3) and immune effector cell-associated neurotoxicity syndrome occurred in 22.9% of patients (7.3% grade ≥ 3). All adverse events were managed effectively. The results demonstrated significant survival benefits with manageable toxicities, supporting tisa-cel as a viable salvage therapy for RR-DLBCL.

**Supplementary Information:**

The online version contains supplementary material available at 10.1007/s12185-025-04006-z.

## Introduction

CD19 chimeric antigen receptor (CAR) T-cell therapies, such as Axicabtagene Ciloleucel, Lisocabtagene maraleucel, and tisagelecleucel, have shown sustained remission and manageable toxicity profiles in patients with relapsed or refractory diffuse large B-cell lymphoma (RR-DLBCL), when used as second- or third-line strategies [1–5]. Since their approval, these CAR-T cell therapies have provided data on long-term outcomes from previously conducted clinical trials, further confirming the efficacy and safety of CAR-T cell treatment in patients with relapsed or refractory diffuse large B-cell lymphoma who were otherwise expected to have dismal survival outcomes [6].

The SCHOLAR-1 study with conventional salvage chemotherapy era reported an overall response rate (ORR) of 26% and median overall survival (OS) of 6.3 months for salvage treatment in RR-DLBCL [6]. Similarly, another prospective cohort study indicated inferior treatment outcomes, with an ORR of 26.4%, a complete response (CR) rate of 9.6%, and a median OS of 7.5 months [7]. Both of these studies presented the limitations of chemotherapy for rescuing RR-DLBCL. Approved CD19 CAR-T cell therapies have demonstrated groundbreaking results, with ORRs ranging from 40 to 70% and median OS exceeding 1 year. However, the excellent treatment outcomes observed are limited by the results that randomized trials have primarily involved patients with RR-DLBCL, with participation restricted to well-controlled subjects selected through stringent enrollment criteria in pivotal clinical studies. Consequently, there is a lack of comprehensive data reflecting the diverse patient characteristics encountered in real-world clinical settings (Table 3). Moreover, the limited real-world experiences reported have often included various B-cell malignancies and results from multiple CAR-T cell therapies analyzed concurrently. These retrospective analyses were conducted based on the complex and diverse characteristics of the patient populations involved, further complicating the interpretation of the findings and not focusing on single CAR-T cell outcomes.

Given these considerations, we analyzed the treatment outcomes and adverse events in 96 patients who received tisa-cel and reported the long-term results of monotherapy for RR-DLBCL.

## Methods

### Study overview

**Fig. 1:**
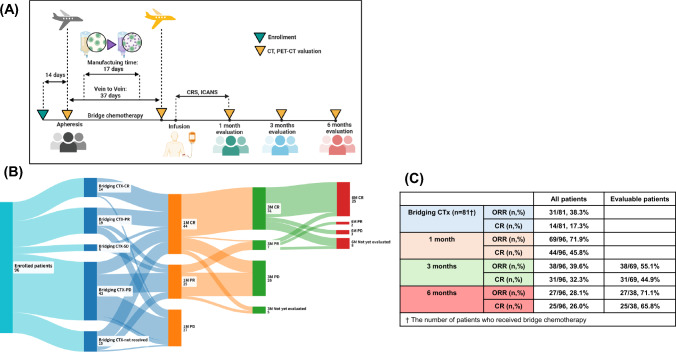
Overview of study **A**, a Sankey diagram illustrating the response rates at enrollment after bridging chemotherapy, and at 1 month, 3 months, and 6 months post-Tisa-cel infusion (**B**), and treatment outcomes following bridging chemotherapy, as well as at 1,3 and 6 months after tisa-cel infusion (**C**)

From 2021 to 2024, we conducted a retrospective study to evaluate the clinical efficacy and safety profile of tisa-cel administered as a third-line or later treatment in patients with RR-DLBCL. The primary objectives were to evaluate the clinical efficacy of tisa-cel in this patient population and analyze the incidence of adverse events and potential safety concerns associated with the therapy during the specified time frame. In Samsung Medical Center (SMC), CAR-T cell therapy requires a median of 14 days from patient registration to the collection of lymphocytes and 37 days from apheresis to the administration of tisa-cel. During this interval, bridging chemotherapy was permitted at the investigator's discretion to manage the disease burden and maintain stability until the tisa-cel infusion was feasible (Fig. 1A).

Clinical data were collected at the time of diagnosis or apheresis, encompassing demographic details (sex, age), Eastern Cooperative Oncology (ECOG-PS), complete blood count (CBC), lactate dehydrogenase (LDH), ferritin levels, C-reactive protein (CRP), serum immunoglobulin IgG level, and disease stage at the diagnosis. Hypogammaglobulinemia was determined based on IgG levels less than 400 mg/dl. Additionally, we documented patients’ prior treatment history, including the number of previous lines of therapy, systemic chemotherapy, autologous hematopoietic stem cell transplantation (ASCT), and bridging therapy preceding tisa-cel. Moreover, we defined the primary refractory, relapsed, or refractory DLBCL. The primary refractory DLBCL was defined as the failure to achieve complete remission (CR) or partial response (PR) after initial frontline treatment or relapse within 12 months of achieving CR [8, 9]. Relapsed DLBCL was referred to patients who had previously responded to treatment (achieved CR or PR), but subsequently experienced disease progression. Refractory DLBCL was defined as failing to respond to the last line of treatment before CAR-T cell therapy [10, 11].

The treatment response evaluation among tisa-cel recipients was performed before and after bridging chemotherapy and 1, 3, and 6 months post-tisa-cel treatment using neck, chest, and abdomen/pelvis computed tomography (CT) scans, as well as 18 F-fluorodeoxyglucose positron emission tomography/computed tomography (FDG PET/CT) scans, following the International Working Group (IWG) response criteria to evaluate treatment efficacy (Fig. 1A) [12–14]. The overall response rate (ORR) for bridging chemotherapy or tisa-cel was determined by the proportion of patients achieving either a CR or PR.

Moreover, CAR-HEMATOTOX, a simple assessable risk-stratification tool for checking hematologic toxicities, was calculated based on laboratory findings before infusion, such as platelet (< 75,000/µl or 75,000–175,000/µl or > 175,000 µL), absolute neutrophil count (ANC; < or > 1200/µL), hemoglobin (Hb; < or > 9/0 g/dL), CRP (< or > 3.0 mg/dL), and ferritin (< 650 ng/mL or 650–2,000ng/mL or > 2000 ng/mL) [15]. Patients receiving CAR-T cell therapy were stratified into two risk groups: a low-risk group (score of 2 or less) and a high-risk group (score of more than 2). The correlation between two risk categories and survival outcomes was analyzed. Based on CAR-HEMATOTOX risk stratification, we assessed the neutropenia (absolute neutrophil count < 500/µL over 14 days), anemia (< 8.0 g/dL), and thrombocytopenia (< 50,000/µL) incidence.

Cytokine release syndrome (CRS) and immune effector cell-associated neurotoxicity syndrome (ICNAS) were defined and assessed under the American Society for Transplantation and Cellular Therapy (ASTCT) guidelines [16]. Additionally, we performed CRS and ICANS management according to the ASTCT guidelines and gathered clinical data regarding the administration of tocilizumab and steroid usage to manage tisa-cel-related adverse events. We assessed the impact of tocilizumab and steroid administration on treatment outcomes and survival rates.

The Institutional Review Board (IRB) of Samsung Medical Center approved this study (IRB 2016-11-040-025) under the ethical principles of the Declaration of Helsinki and the Korea Good Clinical Practice guidelines. Research approval for this study was obtained from the IRB at each participating institution. The final update of the survival data was performed in June 2024.

## Statistical analysis

The significance of differences in categorical variables between groups was assessed using the Chi-square test, with *P*-values less than 0.05 considered significant. The Kaplan–Meier method was used to evaluate the progression-free survival (PFS) and overall survival (OS). PFS was defined as the interval between the date of tisa-cel infusion and the occurrence of either disease progression or all-cause mortality. OS was calculated from the date of tisa-cel infusion to the event of death or last recorded follow-up. Kaplan–Meier curves were generated to make these survival outcomes, and Cox regression analysis was performed to identify prognostic clinical factors associated with PFS and OS. All statistical analyses were conducted using SPSS software, version 25.0 (IBM Corp, Armonk, NY, USA).

## Results

### Baseline characteristics

Of 96 patients, 41 (42.7%) were younger than 60. More patients were male (*n* = 61, 63.5%), and 14 patients (14.6%) presented poor performance status (ECOG-PS ≥ 2). 74.0% of patients (*n* = 71) showed stage III/IV at the diagnosis, and 21.9% (*n* = 21) underwent ASCT. Eighty-one patients (84.4%) received tisa-cel as a third-line treatment following two prior therapeutic regimens. The study included 29 patients (30.2%) who experienced primary refractory and 53 patients (55.2%) who were refractory before receiving tisa-cel. LDH in 69 patients (71.9%) and ferritin in 54 patients (56.8%). Among 81 patients who received bridging therapy, most received a BR (bendamustine and rituximab) regimen (*n* = 48, 46.9%), and 14 patients were administered polatuzumab + BR. Of 19 who received other treatment, 9 underwent platinum-based chemotherapies and 2 received radiotherapy. The outcomes of bridging chemotherapy reported that 33 (40.7%) obtained CR or PR. Additionnaly, we assessed clinical differences between the patients who achieved 3 months CR after tisa-cel and those who did not. The patients who did not achieve 3 months CR presented more refractory (*p* = 0.026), elevated LDH (*p* = 0.030) and poor response after bridging chemotherapy (*p* = 0.002, Table 1).

**Table 1:** Baseline characteristics at the apheresis of the patients who planned tisa-cel

Characteristics	Total patients (*n* = 96)		3 M CR (*n* = 31)		3 M non-CR (*n* = 33)		*p*
	*N*	%	*N*	%	*N*	%	
Age							
< 60 years	41	42.7	16	55.2	13	44.8	0.451
≥ 60 years	55	57.3	15	42.9	20	57.1	
Sex							
Male	61	63.5	17	43.6	22	56.4	0.443
Female	35	36.5	14	56.0	11	44.0	
ECOG-PS							
0–1	82	85.4	29	50.0	29	50.0	0.673
≥ 2	14	14.6	2	33.3	4	66.7	
Stage at the diagnosis							
I/II	25	26.0	7	41.2	10	58.8	0.577
III/IV	71	74.0	24	51.1	23	48.9	
Number of prior CTX							
< 3 lines	81	84.4	27	47.4	30	52.6	0.704
≥ 3 lines	15	15.6	4	57.1	3	42.9	
Prior ASCT							
Received	21	21.9	10	66.7	5	33.3	0.143
Not received	75	78.1	21	42.9	28	57.1	
Disease history							
DLBCL	91	94.8	30	48.4	32	51.6	1.000
tFL	4	4.2	1	50.0	1	50.0	
HGBCL	1	1.0	0		0		
Before status							
Primary refractory	29	30.2	14	70.0	6	30.0	0.026
Relapsed	14	14.6	4	44.4	5	55.6	
Refractory	53	55.2	13	37.1	22	62.9	
LDH							
Elevated	69	71.9	17	38.6	27	61.4	0.030
Normal	27	28.1	14	70.0	6	30.0	
Ferritin (*n* = 95)							
Elevated	54	56.8	17	48.6	18	51.4	1.000
Normal	41	43.2	14	50.0	14	50.0	
CAR-HEMATOTOX							
Low (0–1)	32	33.3	11	45.8	13	54.2	0.800
High (≥ 2)	64	66.7	20	50.0	20	50.0	
Bridging CTX (*n* = 81)							
Pola-BR	14	17.3	4	44.4	5	55.6	0.922
BR	48	59.3	15	48.4	16	51.6	
Others	19	23.5	4	36.4	7	63.6	
Response to bridging CTX (*n* = 81)							
CR+PR	33	40.7	17	68.0	8	32.0	0.002
SD + PD	48	59.3	6	23.1	20	76.9	

*ECOG-PS* Eastern Cooperative Oncology Group Performance Status, *tFL* transformed follicular lymphoma, *HGBCL* high-grade B-cell lymphoma, *CTX* chemotherapy, *ASCT* autologous stem cell transplantation, *pola* polatuzumab, *BR* bendamustine and rituximab, *CR* complete remission, *PR* partial response, *SD* stable disease, *PD* progression disease, *3M* 3 months, *non-CR* non-complete remission

### The response rate from bridging chemotherapy to 6 months after tisa-cel administration

The time from indication discussion to apheresis was 14 days (range 1–58), and vein-to-vein time was calculated as a median of 37 days (range 29–92) (Fig. 1A). Of 96 patients, 81 received bridging chemotherapy, with a response rate of 38.3% (*n* = 31/81) and 17.3% (*n* = 14/81) achieved a CR. Treatment response was evaluated for 96 patients at 1 month. The 1-month ORR for tisa-cel was 71.9% (*n* = 69/96), with a CR rate of 45.8% (*n* = 44/96). The 3-month ORR and CR rates were 39.6% (*n* = 38/96) and 32.3% (31/96), respectively. Among the 69 patients who underwent disease response evaluation at 3 months, the ORR and CR rates were 55.1% (*n* = 38/69) and 44.9% (*n* = 31/69). The 6-month ORR and CR rates were 28.1% (*n* = 27/96) and 26.0% (*n* = 25/96). Among the 38 patients who underwent imaging evaluation at 6 months, the ORR was 71.1% (*n* = 27/38), and the CR rate was 65.8% (*n* = 25/38) (Fig. 1B, C).

### Survival outcomes of tisa-cel

**Fig. 2:**
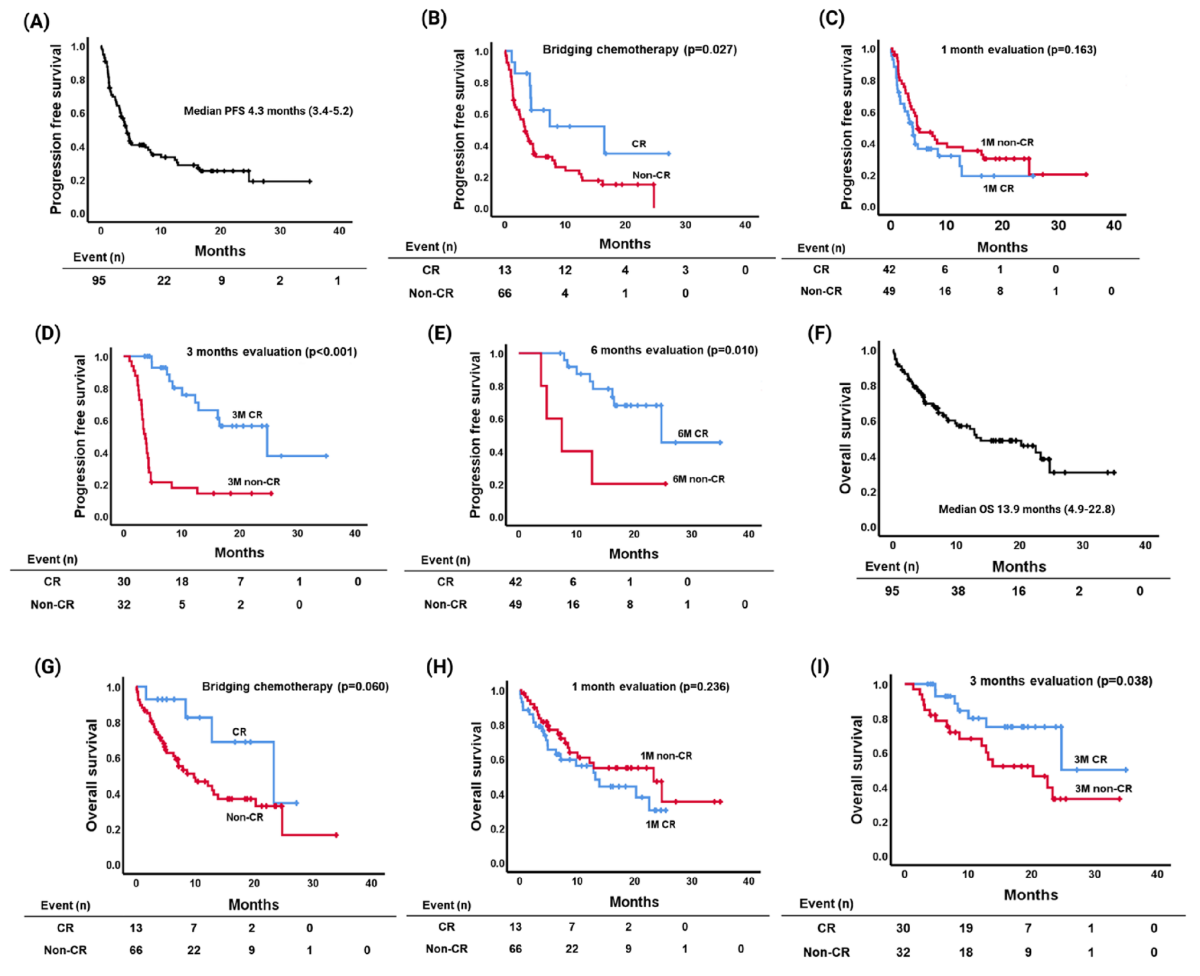
The median progression-free survival (PFS) (**A**), PFS assessment based on bridging chemotherapy response (**B**), PFS assessment at 1-month (**C**), 3 months (**D**), 6 months (**E**) post-tisa-cel treatment; PFS assessment according to HEMATOX MODEL-1 (**F**), the median overall survival (OS) (**G**), OS assessment based on bridging chemotherapy response (**H**), OS assessment at 1 month (**I**), at 3 months (**J**) post-tisa-cel treatment; and OS assessment according to HEMATOX MODEL-1 (**K**)

Over a median follow-up period of 17.3 months, the median PFS was 4.3 months, and 1-year PFS was 33.3% (Fig. 2A). Patients who achieved CR following bridging chemotherapy demonstrated significantly improved PFS compared to those who did not, with a median 16.5 months versus 3.4 months (*p* = 0.027, Fig. 2B). Although there was no significant difference in PFS between patients who achieved CR at 1 month and those who did not (3.9 months vs. 4.8 months, *p* = 0.163, Fig. 2C), patients who achieved CR at 3 months had significantly longer PFS compared to those who did not (24.8 months vs. 3.7 months, *p* < 0.001, Fig. 2D). Moreover, achieving CR at 6 months was associated with superior PFS (24.8 months vs. 7.5 months, *p* = 0.010, Fig. 2E).

The median OS was 13.9 months, and the 1-year OS was 55.2% (Fig. 2F). Patients who achieved CR following bridging chemotherapy had a significantly longer OS compared to those who did not (23.4 months vs. 9.8 months, *p* = 0.060, Fig. 2G), as well as those who achieved CR at 1 month (13.2 months vs. 23.4 months, *p* = 0.236, Fig. 2H). However, patients who achieved CR at 3 months (24.7 months vs. 20.3 months, *p* = 0.038, Fig. 2I) demonstrated extended OS. Through univariate (HR 5.631, 95% CI 2.756–11.505, *p* < 0.001) and multivariate analysis (HR 5.113, 95% CI 2.126–12.297, *p* < 0.001), 3-month CR after tisa-cel infusion was correlated with favorable PFS, although the causality may be vice versa (Table 2). However, multivariate analysis for OS did not reveal any statistically significant prognostic factors.

**Table 2:** Prognostic factors for progression-free survival (PFS) and overall survival (OS) after tisagenlecleucel infusion

	PFS Univariate analysis			PFS Multivariate analysis			OS Univariate analysis		
	HR	95% CI	*p*-value	HR	95% CI	*p*	HR	95% CI	*p*
Age									
< 60 vs. ≥ 60 years	1.550	0.933–2.575	0.090				1.800	0.959–3.378	0.067
Sex									
Male vs. Female	1.019	0.618–1.680	0.942				0.986	0.533–1.822	0.963
ECOG-PS									
0–1 vs. ≥ 2	2.225	1.185–4.178	0.013				3.724	1.869–7.421	< 0.001
Stage at the diagnosis									
I/II vs. III/IV	0.819	0.476–1.409	0.471				0.785	0.412–1.496	0.461
Number of prior CTX									
3 vs. ≥ 4 lines	2.004	1.089–3.689	0.026				1.384	0.972–1.969	0.071
Prior ASCT									
Received vs. not received	1.718	0.876–3.368	0.115				2.651	1.046–6.717	0.040
Before status									
Primary refractory or not	1.487	0.908–2.436	0.115				1.731	0.942–3.181	0.077
Bridging chemotherapy (*n* = 81)									
Pola-BR vs. BR	0.978	0.473–2.070	0.990				1.569	0.590–4.177	0.367
BR ± P vs. Others	0.865	0.643–1.165	0.340				0.949	0.664–1.357	0.774
Response to bridging (*n* = 81)									
CR vs. non-CR	2.369	1.073–5.233	0.033				2.598	0.926–7.291	0.070
LDH									
Normal vs. elevated	1.862	1.029–3.371	0.040				2.074	0.999–4.306	0.050
Ferritin (*n* = 95)									
Normal vs. elevated	0.812	0.495–1.332	0.409				0.609	0.327–1.134	0.118
CRS									
Absence vs. presence	1.110	0.630–1.955	0.719				0.960	0.502–1.836	0.901
ICANS									
Absence vs. presence	1.400	0.820–2.391	0.218				1.502	0.788–2.865	0.217
Tocilizumab use									
Received vs. not received	0.933	0.523–1.664	0.814				1.010	0.518–1.968	0.977
4 vs. over 5 times	1.491	0.800–2.777	0.208				1.777	0.806–3.918	0.154
Steroid use									
Received vs. not received	0.560	0.341–0.921	0.022				0.504	0.277–0.917	0.025
7 vs. over 8 days	1.194	0.577–2.471	0.633				1.694	0.674–4.257	0.262
1-month response after tisa-cel									
CR vs. non-CR	0.701	0.424–1.159	0.166				0.697	0.382–1.270	0.238
3 months response after tisa-cel									
CR vs. non-CR	5.631	2.756–11.505	< 0.001	**5.113**	**2.126–12.297**	** < 0.001**	2.470	1.023–5.966	0.044
Hypogammaglobulinemia									
Absence vs. presence	1.332	0.568–3.124	0.510				1.376	0.354–5.345	0.645

*CTX* chemotherapy, *ASCT* autologous stem cell transplantation, *pola* polatuzumab, *BR* bendamustine and rituximab, *CR* complete remission, *PR* partial response, *SD* stable disease, *PD* progression disease, *ECOG-PS* Eastern Cooperative Oncology Group Performance Status

Bold values indicate statistical significance at *p* < 0.05

According to the records of 36 patients who received follow-up treatment due to disease relapse during the follow-up period, nine patients received lenalidomide or lenalidomide plus rituximab, seven received BR or polatuzumab plus BR, seven participated in a clinical trial, five received immune checkpoint inhibitors such as pembrolizumab or nivolumab, and four underwent cytotoxic chemotherapy. Three were treated with the bispecific antibody (glofitamab) (data was not presented).

### CRS and ICANS after tisa-cel infusion

**Fig. 3:**
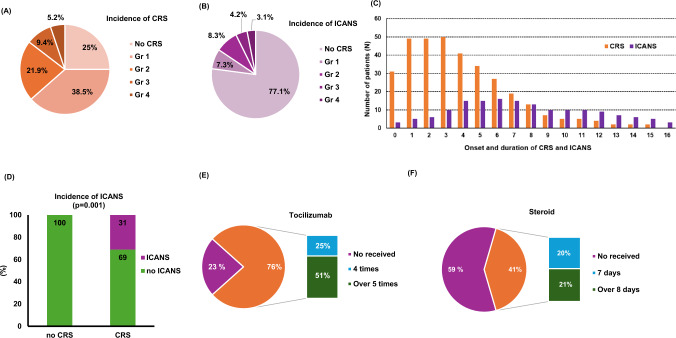
The incidence of CRS (**A**), and ICANS (**B**), the duration of CRS and ICANS (**C**), the correlation between CRS and ICANS (**D**), the frequency of tocilizumab (**E**) and steroid administration (**F**)

75% (*N* = 72) of the patients experienced CRS, with 14.6% experiencing grade 3 or higher CRS events (Fig. 3A). The incidence of ICANS was 22.9%, and 7.3% of the patients experienced grade 3 or higher ICANS (Fig. 3B). The median onset time of CRS was 1 day (range 0–7 days) with a median event duration of 4 days (range 1–16 days). For ICANS, the median onset time was 4 days (range 0–13), and the median duration of events was 5 days (range 1–19 days, Fig. 3C). Notably, no patients developed ICANS only without experiencing CRS, and 31% experienced both CRS and ICANS (*p* = 0.001, Fig. 3D). 76% of patients received tocilizumab to manage CAR-T cell therapy-related side effects, with 25% receiving four doses and 51% receiving five or more doses (Fig. 3E). In addition, 41% of patients required steroid treatment for side effect management; of these, 20% discontinued steroids within 7 days, while 21% received steroids for eight or more days (Fig. 3F). We analyzed the correlation between treatment response rates and the administration of tocilizumab and steroids after tisa-cel infusion. No statistically significant differences in CR rates at 1, 3, and 6 months were observed between patients receiving tocilizumab and steroids. Furthermore, increased doses and prolonged duration of tocilizumab and steroid treatment did not influence treatment response rates in the short or long term (Supplementary Table 1).

**Fig. 4:**
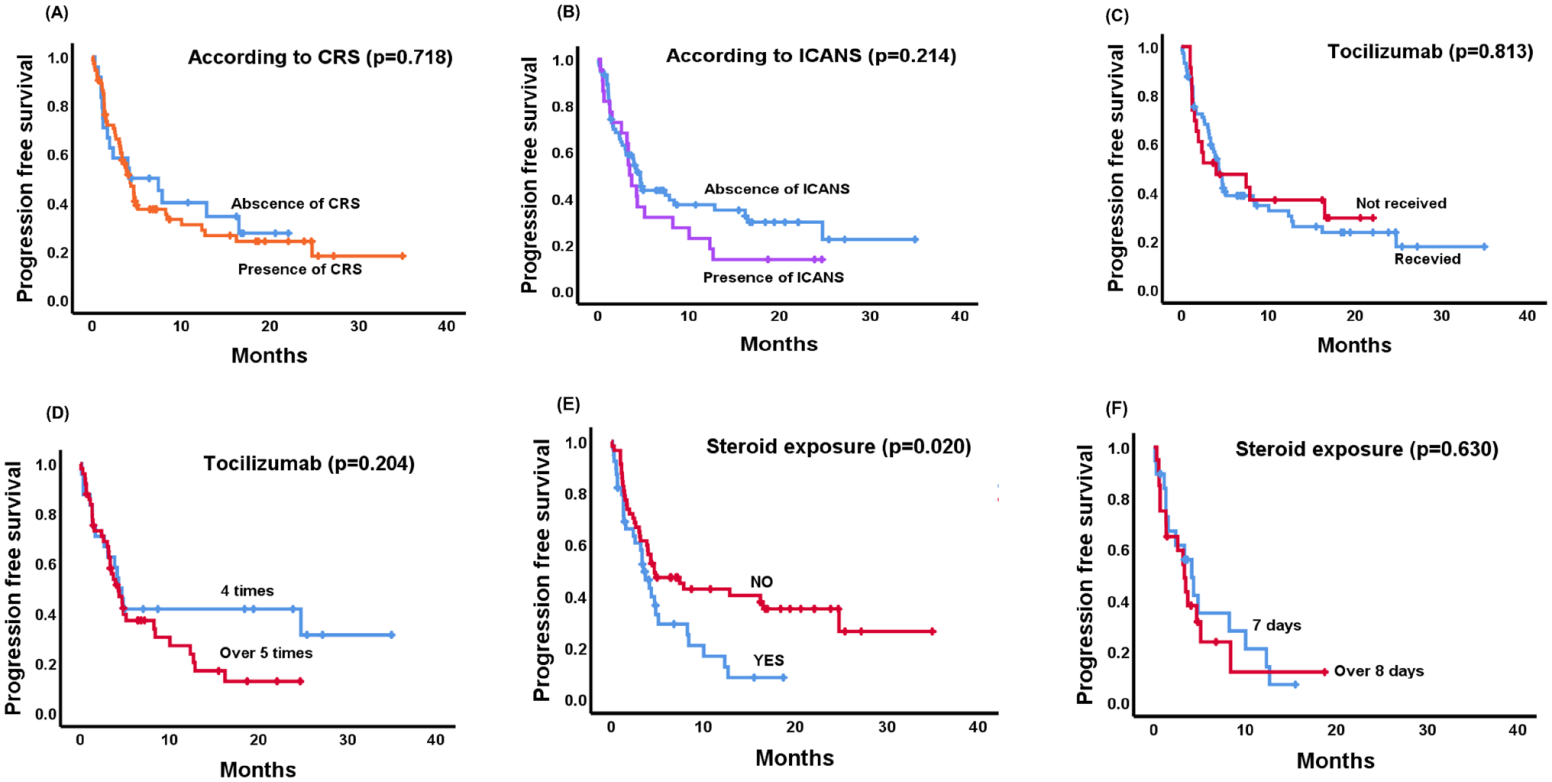
Comparison of PFS between the absence and presence of CRS (**A**), comparison of PFS between patients with and without ICANS (**B**), comparison of PFS between patients who received tocilizumab and those who did not (**C**), comparison of PFS between patients who received tocilizumab four times and those who received it more than five times (**D**), comparison of PFS between patients exposed to steroid and those who were not (**E**), and comparison of PFS between patients who received steroid for 7 days and those who received them for more than 8 days (**F**)

The presence or absence of CRS (4.1 months vs. 4.3 months, *p* = 0.718, Fig. 4A) and ICANS (4.6 months vs. 3.5 months, *p* = 0.214, Fig. 4B) did not influence PFS. There was no statistically significant difference in PFS between patients who received tocilizumab and those who did not (14.2 months vs. 13.3 months, *p* = 0.813, Fig. 4C) unless there was any difference in PFS among patients who received tocilizumab four times versus five or more times (21.1 months vs. 10.0 months, *p* = 0.204, Fig. 4D). Patients who received steroids were reported to have a significantly shorter PFS compared to those who did not (18.1 months vs. 7.3 months, *p* = 0.020, Fig. 4E), although no difference in PFS was observed based on the duration of steroid administration (8.1 months vs. 3.4 months, *p* = 0.630, Fig. 4F).

### The utility of radiotherapy post-CAR-T cell therapy

**Fig. 5:**
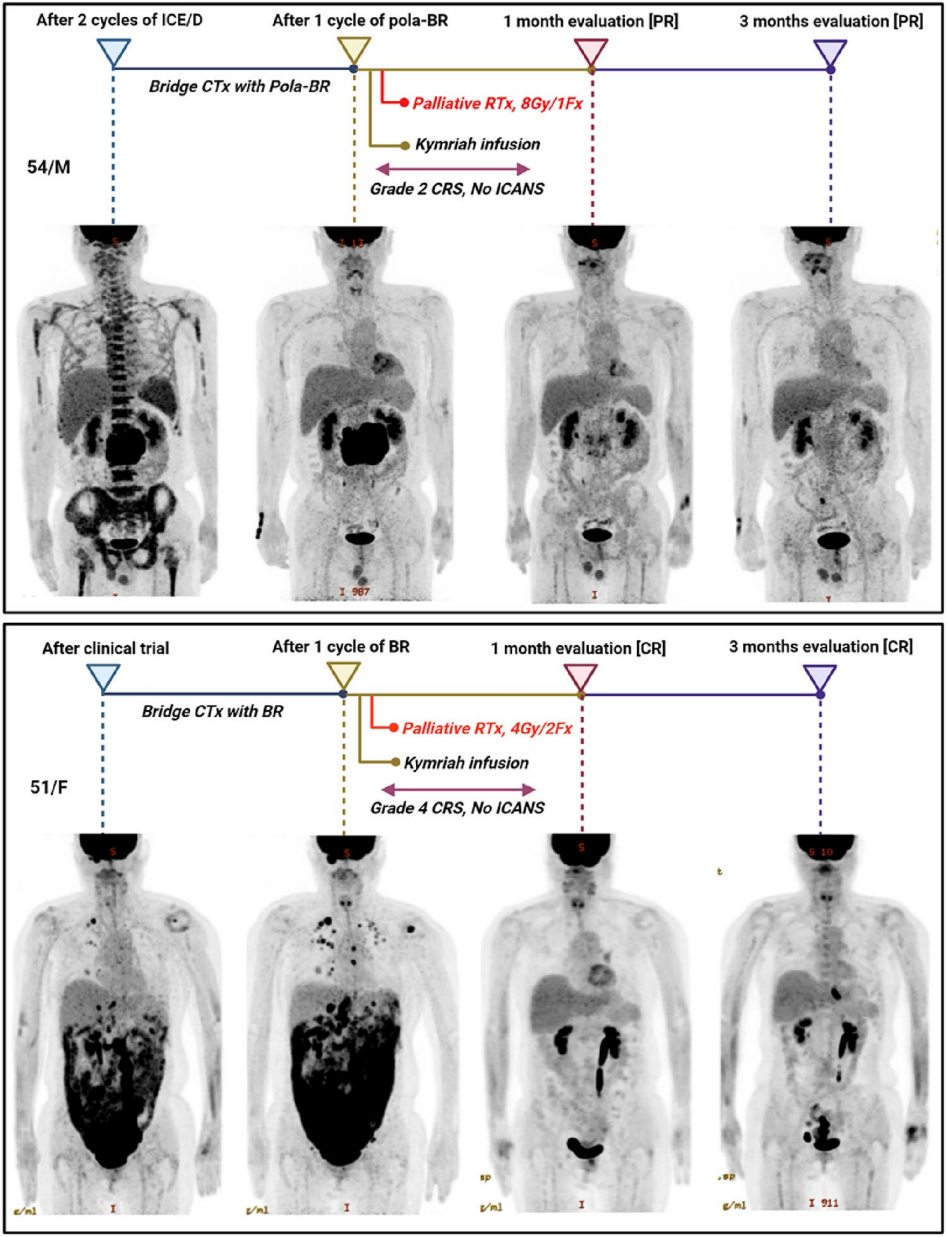
Imaging evaluation of patients who received radiotherapy with tisa-cell infusion (**A**), response evaluation of patients who received radiotherapy after tisa-cel infusion (**B**)

Among the patients who received tisa-cel, we observed that those presenting with large pretreatment masses often expressed concerns regarding the potential for inadequate disease control. To address these concerns, radiotherapy was administered within 2–3 days following the tisa-cel treatment. Remarkably, these patients achieved continuous remission during the 1-month and 3-month disease response evaluations. Furthermore, the management of CRS and ICANS adhered strictly to the ASTCT guidelines, and no additional unexpected side effects were reported, underscoring the safety profile of the treatment (Fig. 5). Additionally, no prolonged cytopenia or unexpected side effects were reported following the conditioning chemotherapy and radiotherapy. This further supports the overall safety and tolerability of the added radiotherapy treatment regimen in this patient population.

### Adverse events related to tisa-cel

Among the total patients treated with tisa-cel, neutropenia occurred in 18 patients (18.8%), anemia presented in 35 patients (63.5%), and thrombocytopenia was documented in 35 patients (36.5%). The patients with a high CAR-HEMATOTOX score showed a higher incidence of neutropenia (*p* = 0.003), anemia (*p* < 0.001), and thrombocytopenia (*p* = 0.002, Supplementary Table 2).

Fifty-three patients checked serum immunoglobulin levels, and 19 (35.8%) experienced hypogammaglobulinemia. Seventeen patients (30.9%) developed infection-related complications and required antibiotics or antiviral agents. Twelve had COVID-19, two had bacterial pneumonia, two had bacteremia, and one had a cytomegalovirus (CMV) infection. No statistically significant associations existed between hypogammaglobulinemia and infection complications (*p* = 0.218). Additionally, intravenous immunoglobulin (IVIG) infusion appeared to have limited effectiveness in preventing patient infection-related complications in this study (*p* = 0.213, Supplementary Table 3).

## Discussion

Since tisa-cel entered the real market, many researchers have expressed concerns about whether its performance in real-world, heterogeneous patient populations would align with the outcomes observed in well-controlled clinical trials. Although the studies reporting real-world data varied in country and patient characteristics, the ORR was 50–70%, and the CR rate was 40–50%, reflecting a trend similar to that observed in the JULIET study (Table 3). Moreover, we demonstrated similar tisa-cel outcomes of 96 RR-DLBCL patients. Therefore, contrary to initial concerns, it has been demonstrated that real-world patients, regardless of country, can achieve improved treatment response rates with tisa-cel than the standard of treatment for RR-DLBCL, comparable to those observed in patients enrolled in well-controlled clinical trials.

**Table 3:** Comparison of CAR-T-cell therapy outcomes in real-world data

References	[1, 30]	Our data	[34]	[35]	[36]	[37]	[38]	[39]
Location	Phase IIJULIET	Korea	U.S	CIBMTR	San Diego	Japan	MD Anderson	Meta-analysis
Type of CAR-T	Tisa-cel	Tisa-cel	NR	Tisa-cel	Tisa-cel or Axi-cel	Tisa-cel	NR	Tisa-cel
Median follow-up	40 months	17.3 months	13.5 months	24 months	16.3 months	6.6 months	11.9 months	16.0 months
Number	93–115 patients	96 patients	97 patients	1159 patients	66 patients (Axi:59,Tisa:7)	89 patients	551 patients	1649 patients
Median age	56 years	63 years	57 years	≥ 65 years: 57.6%	59.5 years	59 years	72 years	64.3 years
Previous Tx	< 3 lines: 49%	Median 2 lines (2–6)	2L: 13% 3L: 39% 4L: 30% 5L + : 19%	≥ 3 lines: 61.0%	Median 3 (range 1–7)	2L: 16.9% 3L: 32.6% 4L: 29.2% 5L + : 21.3%	NR	Median 3 lines (2.6–3.5)
Response rate (%)	53%	71.9%	NR	59.5%	67%	73%	NR	57.7%
CR (%)	39%	45.8%	38%	44.5%	53%	55%	65–69: 34% 70–74: 22% ≥ 75: 29%	39.0%
Median PFS	2.9 months	4.3 months 1-year PFS:33.3%	NR	2-year PFS: 28.4%	10.3 months	1-year EFS: 67.0%	EFS: 7.2 months	3.3 months
Median OS	11 months	13.9 months 1-year OS:55.2%	31.2 months	2-year OS: 43.6%	28.4 months	1-year OS: 46.3%	17.1 months	11.7 months
CRS (all/Gr 3 and 4)	58%/22%	75%/14.6%	NR	58.2%/6.0%	88%/NR	89.9%/6.7%	NR	70.6%/8.9%
ICANS (all/Gr 3 and 4)	21%/12%	22.9%/7.3%	NR	22.5%/7.4%	56%/NR	5.6%/1.1%	NR	19.9%/5.8%

*NR* not reported, *CR* complete response, *Gr* grade, *EFS* event-free survival, *PFS* progression-free survival, *OS* overall survival, *CRS* cytokine release syndrome, *ICANS* immune effector cell-associated neurotoxicity syndrome

In a previously reported study, the time from confirmation of the Tisa-cel indication to apheresis was referred to as"brain to vein,"with an average of 20 days in the USA, 17 days in Europe, and 14 days in this study, suggesting a slightly shorter brain-to-vein time in this study. Additionally, the time from apheresis to Kimriah infusion, termed"vein to vein,"averaged 30 days in the USA, 49 days in Europe, and 37 days at SMC. While no study was analyzed under controlled settings, the average time from indication confirmation to Tisa-cel administration was 50 days in the USA, 66 days in Europe, and 51 days in Korea. The 1-year PFS rates were 31.2% in the USA, 21.3% in Europe, and 33.3% at SMC, while the 1-year OS rates were 71.0%, 42.8%, and 55.2%, respectively. Although direct comparisons are challenging due to differences in study designs, patient characteristics, and regional conditions, the data suggest that a more extended time from collection to administration may impact treatment outcomes. However, using the time consumed before Tisa-cel infusion as a direct prognostic biomarker remains challenging. Further investigation is needed better to understand the relationship between time-dependent factors and Tisa-cel outcomes and to determine how these factors influence treatment efficacy [17]

The incidence of CRS and ICANS, adverse effects associated with CAR-T cell therapy at SMC, was similar to those reported in the Phase II JULIET study and other retrospective studies (Table 3). For mitigating severe symptoms while maintaining treatment efficacy, current guidelines recommend adding dexamethasone to manage any grade CRS that is refractory to at least one dose of tocilizumab, as well as for grade 3 or higher CRS [18–21]. Despite this, there remains considerable concern about the potential reduction in therapeutic response due to steroid use, as some believe that steroids may impact T-cell function, potentially affecting the efficacy of CAR-T cell therapy [22]. According to the ZUMA-1 trial, evaluating the efficacy and safety of axi-cel for RR-DLBCL, the prophylactic and early administration of corticosteroids positively affected overall therapy outcomes [23, 24]. According to our data, all adverse effects during Tisa-cel's treatment were managed with ASTCT guidelines. As shown in Fig. 4E, patients who received steroids had a significantly shorter PFS compared to those who did not. However, this association may be confounded by baseline disease severity rather than a direct effect of steroid use. In particular, elevated LDH levels, an indicator of higher disease burden, were more frequently observed in patients who received steroids. This suggests that steroid use was more common in patients with severe disease, likely due to the need for CRS management rather than being an independent adverse event. Given the high mortality associated with grade 3 or higher CRS and ICANS, timely and adequate steroid administration is expected to improve overall survival outcomes despite its association with shorter PFS.

Regrettably, over 60% of patients experienced disease progression or relapse following CAR-T cell therapy, primarily due to antigen loss on tumor cells, evasion of immune surveillance by tumor, and CAR-T cell exhaustion [25, 26]. According to the DESCAR-T study, the median PFS and OS were reported at 2.8 months and 5.2 months in CAR-T cell therapy failure. However, standardized strategies for addressing CAR-T cell therapy failure have yet to be clearly established. Currently, various therapeutic approaches, including immunomodulatory drugs (e.g., lenalidomide), checkpoint inhibitors (e.g., pembrolizumab), antibody–drug conjugates (e.g., polatuzumab vedotin), bispecific antibodies (e.g., glofitamab, epcoritamab), allogenic stem cell transplantation, and clinical trials are recommended for managing these patients [27, 28]. In patients experiencing cytopenia or slow recovery of health following CAR-T cell therapy, radiation therapy is considered a viable option in patients of local recurrence. In our study, early detection of recurrence was confirmed in two patients, where continuous treatment response was observed without further follow-up interventions following local radiation therapy. Consequently, while local treatment may impose certain burdens on undertreatment, radiation therapy can be a valuable approach to achieve long-term remission, mainly when the recurrence is localized and the patient’s overall condition is compromised.

Although CD19 CAR-T cell therapy brings prolonged survival outcomes and an improved cure rate for RR-DLBCL, it has been reported that more than 50% of nonrelapse mortality in long-term survivors after CAR-T cell therapy is related to infections [29]. The strong ability of anti-CD19 CAR-T cells to target malignant CD19-expressing B cells also destroys normal B cells, often resulting in hypogammaglobulinemia. The highest incidence of hypogammaglobulinemia occurs more than 3 months after CAR-T cell therapy and is directly associated with an increased risk of infection. In the JULIET study, 20% of patients experienced infections within the first 3 months, and the recovery from B-cell aplasia in these patients took over 1 year [30, 31]. Accordingly, many guidelines recommend regular monitoring of serum IgG levels, the proactive administration of intravenous immunoglobulin (IVIG) when serum IgG levels fall below 400 mg/dL, and oral antibiotics and antiviral agents [32, 33]. In this study, there were 46 deaths, with four patients (8.7%) due to COVID-19 infection. 42.9% of the patients had hypogammaglobulinemia at the time of disease response evaluation at 6 months, with a reported infection issue of 53.3% among them (Data was not presented). Although the number of patients analyzed in our study was minimal for checking statistical correlation, making it difficult to establish a clear relationship between hypogammaglobulinemia and infection or between infection and IVIG administration, numerous studies have suggested that mitigating hypogammaglobulinemia may reduce NRM related to infections. As such, efforts to reduce the risk of infection in long-term survivors following CAR-T cell therapy or who failed CAR-T cells and need additional B-cell lymphodepletion treatment remain essential for escaping NRM.

This study has several limitations. First, its retrospective single-center design may introduce selection bias and limit generalizability. Second, the small sample size (*n* = 96) reduces statistical power, necessitating validation in larger, multicenter cohorts. Third, the follow-up duration may not be sufficient to assess long-term survival, subsequent treatment outcomes, and late-onset toxicities, requiring extended observation. Fourth, the impact of steroid use on PFS may be confounded by baseline disease severity, as patients requiring steroids had a higher disease burden. Lastly, the absence of biomarker analyses, such as CAR-T cell expansion, persistence, and tumor microenvironmental factors, limits mechanistic insights into treatment response and resistance. Future studies should address these limitations by incorporating larger sample sizes, extended follow-up periods, and comprehensive biological assessments to optimize patient selection and therapeutic strategies.

In conclusion, Tisa-cel has demonstrated comparable therapeutic outcomes in a heterogeneous patient population in real-world settings without exceeding the established safety profile, as demonstrated in a well-controlled clinical study. Consequently, Tisa-cel could recommended as a third-line treatment option, as it offers a median overall survival (OS) of 14 months in patients with relapsed or refractory diffuse large B-cell lymphoma (RR-DLBCL), compared to the expected OS of 6–7 months with alternative therapies. Concerning short-term side effects such as CRS and ICANS, the administration of steroids and tocilizumab does not appear to affect therapeutic outcomes or survival adversely, so proactive management is warranted. Furthermore, ongoing patient monitoring and the use of IVIG are recommended to address potential long-term infection-related issues.

## Supplementary Information

Below is the link to the electronic supplementary material.

Supplementary file1 (DOCX 22 KB)

## Data Availability

All data will become publicly available upon request to the corresponding authors.
