# Supplementary material for: Clinical outcomes of tisagenlecleucel in relapsed/refractory diffuse large B-cell lymphoma: insights from a single-center study
Source: Int J Hematol. 2025 Aug 22;122(4):533–45. doi: 10.1007/s12185-025-04006-z (PMC12476396; doi:10.1007/s12185-025-04006-z)
Supplement: Supplementary file 1 — Supplementary file1 (DOCX 22 KB) [file 12185_2025_4006_MOESM1_ESM.docx]

**Supplementary Table S1. Tocilizumab and steroid administration according to timeline**

**.**

|  |  | 1M CR | 1M  non-CR | P-value | 3M CR | 3M  non-CR | P-value | 6M CR | 6M  non-CR | P-value |
| --- | --- | --- | --- | --- | --- | --- | --- | --- | --- | --- |
| Tocilizumab | Received | 42.9% | 57.1% | 0.336 | 43.8% | 56.3% | 0.252 | 81.0% | 56.7% | 1.000 |
|  | Not received | 56.5% | 43.5% |  | 62.5% | 37.5% |  | 88.9% | 26.7% |  |
| Tocilizumab | 4 times | 39.1% | 60.9% | 0.798 | 43.8% | 56.3% | 1.000 | 77.8% | 22.2% | 1.000 |
|  | ≥ 5 times | 44.7% | 55.3% |  | 43.8% | 56.3% |  | 83.3% | 16.7% |  |
| Steroid | Received | 41.7% | 58.3% | 0.527 | 38.1% | 61.9% | 0.294 | 71.4% | 28.6% | 0.565 |
|  | Not received | 49.1% | 50.9% |  | 53.5% | 46.5% |  | 87.0% | 13.0% |  |
| Steroid | 7 days | 42.1% | 57.9% | 1.000 | 36.4% | 63.6% | 1.000 | 60.0% | 40.0% | 0.524 |
|  | ≥ 8 days | 41.2% | 58.8% |  | 40.0% | 60.0% |  | 100.0% | 0.0% |  |

**Supplementary Table S2. Hematologic toxicities**

|  | Total patients | CAR-HEMATOTOX | | |
| --- | --- | --- | --- | --- |
|  |  | Low | High | P-value |
| Neutropenia (<500/uL) over 14 days | 18 (18.8%) | 1 (5.6%) | 17 (94.4%) | 0.003 |
| Anemia (<8.0g/dL) | 35 (36.5%) | 4 (12.5%) | 31 (88.6%) | <0.001 |
| Thrombocytopenia  (<50,000/uL) | 35 (36.5%) | 5 (15.6%) | 30 (85.7%) | 0.002 |

**Supplementary Table S3. The incidence of hypogammaglobulinemia and infection.**

| Total patients | Hypogammaglobulinemia | Infection | IVIG infusion |
| --- | --- | --- | --- |
| Evaluation patients | 53 | 55 | 56 |
| Presence | 19 (35.8%) | 17 *(30.9%) | 18 (32.1%) |
| Absence | 34 (64.2%) | 38 (69.1%) | 37 (67.9%) |
| P-value | 0.218 | |  |
|  |  | 0.213 | |

*** 17 infection issues: 12 COVID19, 2 bacterial pneumonia, 2 bacteremia, 1 CMV colitis**
